# Association of underweight status with the risk of tuberculosis: a nationwide population-based cohort study

**DOI:** 10.1038/s41598-022-20550-8

**Published:** 2022-09-28

**Authors:** Su Hwan Cho, Hyun Lee, Hyuktae Kwon, Dong Wook Shin, Hee-Kyung Joh, Kyungdo Han, Jin Ho Park, Belong Cho

**Affiliations:** 1 Department of Family Medicine, Seoul National University Hospital, Seoul, Republic of Korea; 2 Department of Internal Medicine, Hanyang University College of Medicine, Seoul, Republic of Korea; 3 Center for Health Promotion and Optimal Aging, Seoul National University Hospital, Seoul, Republic of Korea; 4 Department of Family Medicine/Supportive Care Center, Samsung Medical Centre, Sungkyunkwan University School of Medicine, Seoul, Republic of Korea; 5 Department of Medicine, Seoul National University College of Medicine, Seoul, Republic of Korea; 6 Department of Family Medicine, Seoul National University Health Service Center, Seoul, Republic of Korea; 7 Department of Statistics and Actuarial Science, Soongsil University, Seoul, Republic of Korea; 8 Department of Family Medicine, Seoul National University College of Medicine, Seoul, Republic of Korea

**Keywords:** Diseases, Risk factors

## Abstract

In studies evaluating the association between body mass index (BMI) and risk of tuberculosis (TB), the data for the underweight population has been limited and results were conflicting. Our study aimed to evaluate whether being underweight increases the risk of TB using a nationwide representative sample from the Republic of Korea. A large population-based cohort study of over ten million subjects who participated in the health screening in 2010 was performed using the Korean National Health Insurance database 2010–2017. We evaluated the incidence and risk of TB by BMI category (kg/m^2^) for Asians using a multivariable Cox regression model, adjusting for age, sex, smoking, alcohol consumption, regular exercise, low-income state, and underlying hypertension, diabetes mellitus, and dyslipidemia. To evaluate the association between BMI and TB risk, the underweight population was further subdivided according to the degree of thinness. During 70,063,154.3 person-years of follow-up, 52,615 of 11,135,332 individuals developed active TB with an incidence of 0.75 per 1000 person-years. Overall, there was a log-linear inverse relationship between TB incidence and BMI, within the BMI range of 15–30 kg/m^2^ (R^2^ = 0.95). The estimated adjusted hazard ratio (HR) for incident TB in the underweight population (BMI < 18.5) was 2.08 (95% confidence intervals, CI 2.02–2.15), overweight (23 ≤ BMI < 25) was 0.56 (0.55–0.58) and obese (BMI ≥ 25) was 0.40 (0.39–0.41) relative to the normal weight population. Among the underweight population, TB risk increased as the degree of thinness increased (adjusted HR = 1.98, 1.91–2.05; 2.50, 2.33–2.68; and 2.83, 2.55–3.15, for mild, moderate and severe thinness, respectively) (*p* for trend < 0.001). We found a significant inverse relationship between BMI and TB incidence, which was especially profound in the underweight population. Public health strategies to screen TB more actively in the underweight population and improve their weight status may help reduce the burden of TB.

## Introduction

Despite worldwide efforts to reduce the global burden of tuberculosis (TB), TB is still a significant threat to global health, reaching one of the top causes of death worldwide. Several underlying diseases^1,2^, lifestyle habits^3^, and social determinants^4^ are known to contribute to the development of tuberculosis. Among them, undernutrition is known to attribute to new TB cases the most^5^. Since body mass index (BMI) is most widely used as an indicator of nutritional state, there have been many studies attempting to clarify the association between an underweight state and the prevalence or incidence of TB^6–8^.

However, since most of the previous studies used a cross-sectional design, the association of an underweight state with the incidence of TB was not clearly shown^9^. One recent systemic review has overcome this limitation by analyzing six cohort studies and highlighted that lower BMI was associated with an increased risk of TB^10^. However, most studies enrolled in this review were not based on the general population^11–15^. Moreover, as data on the underweight subjects were not sufficient, it was not clear whether a severe underweight state further increases TB risk^10^. Conversely, a recent cohort study performed in Taiwan showed conflicting results that an underweight state was not associated with an increased risk of TB^16^. Thus, a well-designed nationwide cohort study with sufficient underweight participants is needed to confirm whether low BMI increases the risk of TB. In addition, it is necessary to determine whether the underweight state is associated with the incidence of TB in high-income countries, unlike countries with the highest incidence of TB.

Therefore, our study aimed to evaluate whether an underweight state, measured by BMI, increases the risk of TB using a nationwide representative sample from the Republic of Korea. We further evaluated whether the risk of TB increases as BMI decreases even within an underweight group.

## Methods

### Study hypothesis

We hypothesized that the underweight population would have an increased incidence of TB compared to the normal weight population by the deficient state of the immune system due to undernutrition^17^. Also, the degree of immunodeficiency induced by the lower body weight would have shown a dose–response relationship even after adjusting for the effects of other confounding variables.

### Data source and study population

The National Health Information Database (NHID) is a public database established by the Korean National Health Insurance Service (NHIS), which is mandatory for the entire population of the Republic of Korea (above 50 million). The NHID consists of the following databases: the eligibility database, which includes sociodemographic variables, the health care utilization database, which includes information on medical usage and prescription records, the long-term care insurance database, the health care provider database and the national health screening database^18^. The NHIS provides a national health screening program at least biennially for employees of all ages and biennially for those older than 40 years. This program consists of a survey about health behaviors, medical history and family history, anthropometric measurements and laboratory tests^19^.

**Figure 1:**
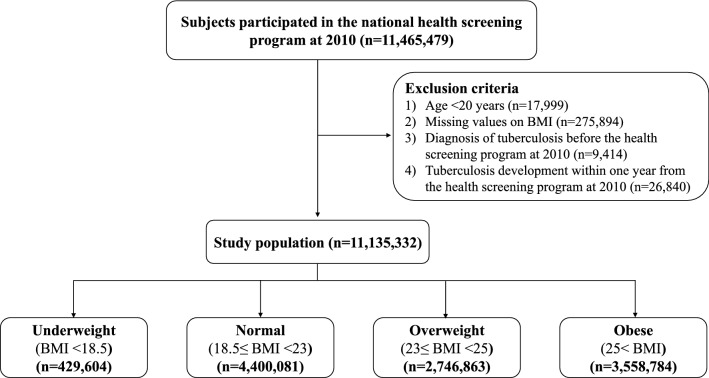
Flow chart of the study population.

We collected data from the NHID for all individuals who participated in the national health screening program in 2010. Among 11,465,479 participants, we excluded 17,999 individuals under 20 years old, 275,894 individuals with missing variables and 9414 individuals who had received a diagnosis of TB before the date of the program in 2010. In addition, we excluded 26,840 individuals who developed TB within 1 year from the date of the health screening to minimize reverse causality. The final study population consisted of 11,135,332 individuals (Fig. 1). All participants were followed up from the date of the national health screening in 2010 to the date of TB diagnosis or December 31, 2017, whichever came first.

The Seoul National University Hospital (Seoul, Republic of Korea) institutional review board approved this study (IRB no. E-1903-010-1014) and waived the requirement for written informed consent because NHID provides anonymized and de-identified data. All research was performed following the 1964 Declaration of Helsinki and its later amendments.

### Outcome and covariable definitions

There is a specific code system to reduce the copayment of patients diagnosed with rare intractable diseases in the Korean NHIS. Disease-specific confirmation results and doctor’s diagnoses are required to be registered in this code system. TB was defined with the specific codes V246, V206 and V101 until June 2016, and V000 thereafter which correspond to the TB of the International Classification of Diseases, 10th revision codes (ICD-10)^19^.

Participants were categorized by BMI (kg/m^2^) using the WHO Western Pacific Region guideline strata of underweight (< 18.5), normal weight (18.5–22.9), overweight (23.0–24.9), and obesity (≥ 25.0)^20^. The underweight population was further categorized into mild (17.0–18.4), moderate (16.0–16.9), and severe (< 16.0) thinness^21^.

Study subjects submitted responses to questionnaires regarding their past medical history and health behaviors such as smoking, alcohol consumption and physical activities at the national health screening program. Smoking status was categorized into never smoker, ex-smoker, or current smoker. Alcohol consumption status was categorized into non-drinker, mild drinker (< 30 g/day of alcohol), and heavy drinker (≥ 30 g/day of alcohol)^22^. We defined participants as regular exercisers if they participated in vigorous activity (≥ 20 min/day) three or more times a week or moderate activity (≥ 30 min/day) five or more times a week^23^.

Comorbidities such as hypertension, diabetes, and dyslipidemia were defined by prescription of medication for the disease using the ICD-10 codes (I10-13 and I15 for hypertension; E11-14 for diabetes; and E78 for dyslipidemia) before health screening or results of the program show that the diagnostic criteria were met (systolic blood pressure ≥ 140 mmHg or diastolic blood pressure ≥ 90 mmHg for hypertension^24^; fasting blood glucose ≥ 126 mg/dL for diabetes^25^; total cholesterol ≥ 240 mg/dL for dyslipidemia^26^). Because the health insurance premiums are determined by income level in the NHIS, we defined the low-income population as individuals whose health insurance premiums were less than the lowest quintile in the insured or who were medical aid beneficiaries.

### Statistical analysis

The baseline characteristics of participants were presented as mean (standard deviation) or numbers (%) according to BMI categories. TB incidence rates were calculated by dividing the number of events by 100,000 person-years (PY). Then, the TB incidence rate was fitted by a linear model for BMI on a logarithmic scale to examine the association for the range of BMI 15–30. Cox proportional hazards regression analyses were conducted to obtain the hazard ratios (HRs) and 95% confidence intervals (CIs) of TB based on whole BMI categories and especially in the underweight subpopulation. The risk of TB was analyzed after adjustments for possible factors. Model 1 was adjusted for age and sex, and model 2 was additionally adjusted for lifestyle (smoking status, alcohol consumption and physical activity) and low-income state. Model 3 was further adjusted for history of hypertension, diabetes and dyslipidemia. Stratified analysis was performed by dividing the participants into subgroups according to age (< 40, 40–65, or ≥ 65 years), sex, smoking status, alcohol consumption, physical activity, income and presence of hypertension, diabetes, and dyslipidemia. Statistical analyses were conducted using SAS (version 9.4; SAS Institute, Cary, NC, US) and STATA software (MP, version 16.1; StataCorp, College Station, TX, US), and statistical significance was defined as two-sided *p* < 0.05.

## Results

### Baseline characteristics of subjects

As shown in Table 1, the underweight population was more likely to be younger (mean 40.8 years old) and included more women (69.3%). The underweight population had a higher rate of never-smokers (72.8%) and non-drinkers (59.0%) compared to other populations (59.3% for never-smokers and 51% for non-drinkers). The rate of subjects who regularly exercise was lower in the underweight population (9.9%) than in other populations. The underweight population had fewer comorbidities, such as hypertension (9.7%), diabetes mellitus (3.3%), and dyslipidemia (5.6%), compared to other populations (27.1% for hypertension, 8.9% for diabetes mellitus, and 18.7% for dyslipidemia).

**Table 1:** Baseline characteristics of the study population.

	Underweight (BMI < 18.5 kg/m^2^) (n = 429,604)	Normal weight (18.5 ≤ BMI < 23.0 kg/m^2^) (n = 4,400,081)	Overweight (23.0 ≤ BMI < 25.0 kg/m^2^) (n = 2,746,863)	Obesity (BMI ≥ 25.0 kg/m^2^) (n = 3,558,784)	*p* value
**Age, years**	40.8 (16.2)	45.7 (14.2)	49.1 (13.2)	49.1 (13.2)	< 0.01
**Age group**					< 0.01
< 40	245,132 (57.0)	1,556,970 (35.4)	673,023 (24.5)	906,373 (25.5)	
40–64	132,610 (30.9)	2,341,268 (53.2)	1,697,747 (61.8)	2,152,253 (60.5)	
> 65	51,862 (12.1)	501,843 (11.4)	376,093 (13.7)	500,158 (14.0)	
**Sex**					< 0.01
Male	131,953 (30.7)	2,035,688 (46.3)	1,658,038 (60.4)	2,258,794 (63.5)	
Female	297,651 (69.3)	2,364,393 (53.7)	1,088,825 (39.6)	1,299,990 (36.5)	
**Smoking status**					< 0.01
Never	312,775 (72.8)	2,867,018 (65.2)	1,564,347 (57.0)	1,917,620 (53.9)	
Ex-smoker	27,915 (6.5)	508,402 (11.5)	484,351 (17.6)	669,729 (18.8)	
Current smoker	88,914 (20.7)	1,024,661 (23.3)	698,165 (25.4)	971,435 (27.3)	
Alcohol consumption*					< 0.01
Non-drinker	253,682 (59.0)	2,390,351 (54.3)	1,376,814 (50.1)	1,735,101 (48.7)	
Mild drinker	158,894 (37.0)	1,752,072 (39.8)	1,151,854 (41.9)	1,471,783 (41.4)	
Heavy drinker	17,028 (4.0)	257,658 (5.9)	218,195 (8.0)	351,900 (9.9)	
**Regular exercise**^**†**^	42,578 (9.9)	752,908 (17.1)	570,476 (20.8)	721,697 (20.3)	< 0.01
**Low-income**^**††**^	81,381 (18.9)	856,556 (19.5)	520,248 (18.9)	675,597 (19.0)	< 0.01
**Comorbidities**					
Hypertension	41,583 (9.7)	740,091 (16.8)	761,864 (27.7)	1,400,123 (39.3)	< 0.01
Diabetes mellitus	14,114 (3.3)	244,807 (5.6)	247,933 (9.0)	459,685 (12.9)	< 0.01
Dyslipidemia	23,985 (5.6)	533,376 (12.1)	543,863 (19.8)	925,269 (26.0)	< 0.01

Data are presented as the mean (SD) and number (%)

*BMI* body mass index.

*Alcohol consumption was classified into mild drinker (less than 30 g/day of alcohol) and heavy drinker (30 g/day or more than 30 g/day of alcohol).

^†^Regular exerciser was defined as individuals who did vigorous activity (> 20 min/day) at least three times per week or moderate activity (> 30 min/day) at least five times per week.

^††^Low-income participants were defined as health insurance premiums less than the lowest quintile in the insured or medical aid beneficiaries.

### Incidence and risk of TB according to BMI category

**Figure 2:**
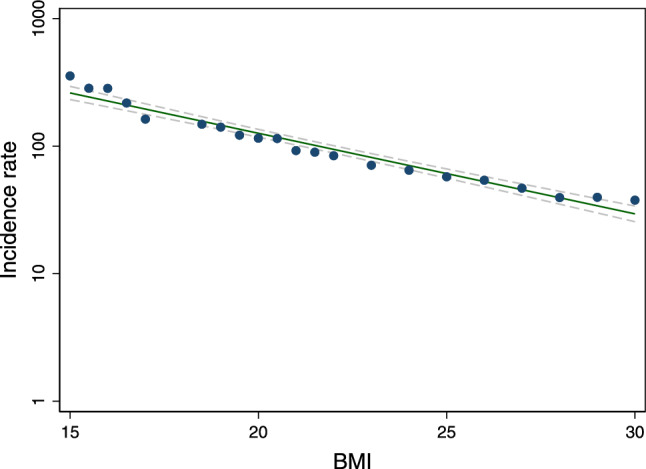
The inverse logarithmic relationship between body mass index and tuberculosis incidence rate in the study population The incidence rate was fitted by a linear model for body mass index (BMI) on a logarithmic scale to examine the association for the range of BMI 15–30. (R^2^ = 0.95).

During 70,063,154.3 PY of follow-up, 52,615 of 11,135,332 subjects developed TB with an incidence of 0.75 per 1000 PY. Overall, there was a log-linear inverse relationship between TB incidence and BMI, within the BMI range of 15–30 kg/m^2^ (R^2^ = 0.95) (Fig. 2). The incidence and risk of TB by BMI category are summarized in Table 2. The incidence of TB (per 1000 PY) increased as BMI decreased **(**1.71 for underweight, 0.97 for normal weight, 0.63 for overweight, and 0.46 for obese populations). The estimated HR for incident TB in the underweight population was significantly increased when the normal weight population was referenced (adjusted HR in the fully adjusted model = 2.08, 95% CI 2.02–2.15). By contrast, the estimated HR for incident TB was inversely associated with increased BMI (adjusted HR = 0.56, 95% CI 0.55–0.58 for overweight population and adjusted HR = 0.40, 95% CI 0.39–0.41 for obese population; p for trend < 0.01).

**Table 2:** The incidence and risk of tuberculosis by BMI category.

BMI category (kg/m^2^)	Number at risk	TB events	Person-years	Incidence rate (per 1000 person-year)	Estimated hazard ratio (95% confidence interval)			
					Non-adjusted	Model 1*	Model 2^†^	Model 3^‡^
< 18.5	429,604	4549	2,657,961.15	1.71	1.77 (1.72, 1.83)	2.01 (2.02, 2.15)	2.05 (1.98, 2.11)	2.08 (2.02, 2.15)
18.50–22.9	4,400,081	26,710	27,637,461.41	0.97	Reference	Reference	Reference	Reference
23.00–24.9	2,746,863	10,976	17,321,680.31	0.63	0.66 (0.64, 0.67)	0.57 (0.56, 0.58)	0.57 (0.56, 0.59)	0.56 (0.55, 0.58)
≥ 25.0	3,558,784	10,380	22,446,051.46	0.46	0.48 (0.47, 0.49)	0.41 (0.40, 0.42)	0.42 (0.41, 0.42)	0.40 (0.39, 0.41)
*p* for trend					< 0.001	< 0.001	< 0.001	< 0.001

*BMI* body mass index.

*Adjusted for age and sex.

^†^Adjusted for variables in model 1, smoking, alcohol consumption, regular exercise and low-income status.

^‡^Adjusted for variables in model 2, hypertension, diabetes mellitus and dyslipidemia.

### Incidence and risk of TB among the underweight subpopulation by BMI category

The incidence and risk of TB by BMI category in the underweight population are summarized in Table 3. Among the underweight population, the incidence of TB increased as the degree of thinness increased (0.97 for normal weight, 1.57 for mild thinness, 2.09 for moderate thinness, and 3.31 for severe thinness per 1000 PY).

**Table 3:** Incidence and risk of tuberculosis in the underweight subpopulation by BMI category.

BMI category (kg/m^2^)	Number at risk	TB Events	Person-years	Incidence rate (per 1000 person-year)	Estimated hazard ratio (95% confidence interval)			
					Crude model	Model 1*	Model 2^†^	Model 3^‡^
Normal (18.5 ≤ BMI < 23.0)	4,400,081	26,710	27,637,461.41	0.97	Reference	Reference	Reference	Reference
Mild thinness (17.0 ≤ BMI < 18.5)	349,652	3406	2,173,728.20	1.57	1.62 (1.57, 1.68)	2.00 (1.93, 2.07)	1.97 (1.90, 2.04)	1.98 (1.91, 2.05)
Moderate thinness (16.0 ≤ BMI < 17.0)	61,445	786	376,382.18	2.09	2.16 (2.01, 2.32)	2.55 (2.37, 2.74)	2.50 (2.33, 2.69)	2.50 (2.33, 2.68)
Severe thinness (BMI < 16.0)	18,507	357	107,850.76	3.31	3.42 (3.09, 3.80)	2.72 (2.44, 3.02)	2.67 (2.40, 2.97)	2.83 (2.55, 3.15)
*p* for trend					< 0.001	< 0.001	< 0.001	< 0.001

*BMI* body mass index.

*Adjusted for age and sex.

^†^Adjusted for variables in model 1, smoking, alcohol consumption, regular exercise and low-income status.

^‡^Adjusted for variables in model 2, hypertension, diabetes mellitus and dyslipidemia.

**Figure 3:**
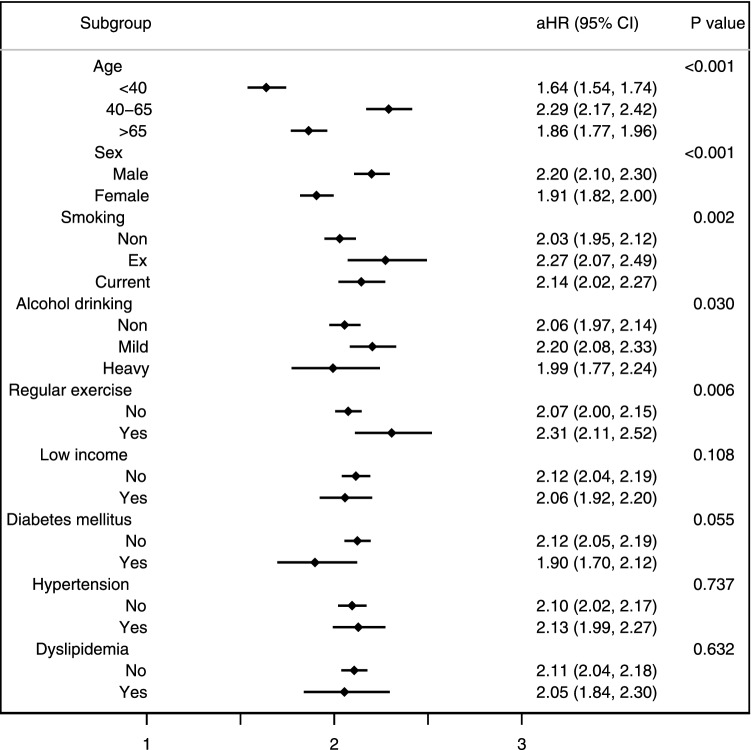
Subgroup analysis of adjusted hazard ratio for TB in the underweight population.

Across the BMI category in the underweight subpopulation, a significant inverse correlation between BMI and TB risk was observed (*p* for trend < 0.001). The estimated adjusted HR for incident TB in the underweight population relative to normal weight population increased as the degree of thinness increased (adjusted HR = 1·98, 95% CI 1.91–2.05 for mild thinness; adjusted HR = 2.50, 95% CI 2.33–2.68 for moderate thinness; adjusted HR = 2.83, 95% CI 2.55–3.15 for severe thinness; *p* for trend < 0.001). The negative association between BMI category and incident TB among the underweight population was consistent in all subgroup analyses (Fig. 3).

## Discussion

In this large nationwide observational study, BMI is significantly associated with an increased risk of incident TB in an inverse logarithmic relationship and further showed that this trend was maintained even when the underweight population was subdivided. Compared to normal weight subjects, those with mild, moderate, and severe thinness had 2.0, 2.5, and 2.8-fold increased risk of TB, respectively.

In agreement with previous findings showing an inverse relationship between TB risk and BMI^10^, our study showed that BMI increases the risk of TB. Our study has the advantage of extending these findings to the underweight population. Due to the insufficient number of underweight participants, the previous studies did not confirm the inverse relationship between BMI and TB risk in the underweight population^11–15^. Since our study was a nationwide population-based study and included enough underweight individuals, our findings have provided more robust evidence of the association between the underweight state and TB incidence in the general population.

Generally, the underweight state (BMI < 18.5 kg/m^2^) is considered to represent undernutrition in adults, although this anthropometric measure does not fully reflect all nutrient deficiencies^27^. Undernutrition represented by protein and calorie deficiencies decreases the efficiency of the cell-mediated immunity system and several innate host defense mechanisms which can be used to defend against TB^28^. As a result, protein-calorie malnutrition induces a secondary immunodeficiency state which increases the risk of developing TB^17^. Our study found that the incidence of TB increased as the degree of underweight became more severe, suggesting that the worsening of nutritional deficiencies further exacerbates this secondary immunodeficiency. These findings suggest that the improvement in public nutritional status may be essential across the public health strategies for TB from prevention to treatment. The strategy to reduce TB burden using nutritional support might have other advantages in that it might modify the natural course of TB. In addition, it is known that undernutrition could be associated with the severity of TB^29,30^, poor treatment outcomes, including a longer time to sputum conversion^31^, and higher mortality^32–34^. In addition, failure to improve weight gain during TB treatment is associated with relapse during treatment^35^.

Thus, in our study, the evaluation of the relationship between BMI and TB risk among the underweight population in the Republic of Korea is noteworthy. As the BMI category changed from mild thinness to severe thinness, the risk of TB increased in a weight-dependent manner. These results suggest that the key solution to controlling TB is in improving public nutritional status. A previous study also supports this view by showing only a small improvement in nutrition could have a substantial impact on the incidence of TB in areas with a high prevalence of undernutrition^36^. However, not only nutritional status but also other factors can be related to being underweight (e.g., low birth weight, respiratory infection in childhood, etc.)^37,38^. Thus, in some individuals, low BMI might be merely reflective of other conditions. Unfortunately, due to the lack of information, we could not evaluate whether lower BMI increases the risk of TB independently of these factors. Further studies are needed on this issue.

### Limitation

There are some important limitations to our study. First, the specific code for TB used in this study included all types of TB, so a stratified analysis according to the type of TB was not possible. Second, because this study was conducted with a population of a high-income country in Asia, it might not be generalized to situations in other countries or ethnic groups with different environments. However, as the environmental factors affecting BMI in high-income countries are expected to be lower than in low-income countries, our study may have an advantage in that we could have evaluated the impact of being underweight on the risk of TB more accurately. Additionally, using representative nationwide data in a single country can also be the advantage of our study, as many previous studies have evaluated that the relationship between BMI and TB risk was not free from selection bias.

## Conclusion

In conclusion, the severity of being underweight as defined by BMI was associated with a higher risk of TB incidence in a weight-dependent manner, even in an underweight population. Strengthening TB screening programs for the underweight population to detect TB patients earlier may help reduce the burden of TB.

## Data Availability

The data that support the findings of this study are available from National Health Information Database but restrictions apply to the availability of these data, which were used under license for the current study, and so are not publicly available. Data are however available from the authors upon reasonable request and with permission of National Health Insurance Sharing Service (https://nhiss.nhis.or.kr). For further information regarding data availability, please contact the corresponding author, Jin Ho Park. (pjhn@snu.ac.kr).

## Abbreviations

BMI: Body mass index
CI: Confidence interval
HR: Hazard ratio
ICD-10: The international classification of diseases, 10th revision codes
NHID: The National Health Information Database
NHIS: The Korean National Health Insurance Service
PY: Person-years
TB: Tuberculosis
